# High resolution profiling of human exon methylation by liquid hybridization capture-based bisulfite sequencing

**DOI:** 10.1186/1471-2164-12-597

**Published:** 2011-12-08

**Authors:** Junwen Wang, Hui Jiang, Guanyu Ji, Fei Gao, Mingzhi Wu, Jihua Sun, Huijuan Luo, Jinghua Wu, Renhua Wu, Xiuqing Zhang

**Affiliations:** 1 Beijing Genomics Institute at Shenzhen, Beishan Road, Shenzhen 518000, China

## Abstract

**Background:**

DNA methylation plays important roles in gene regulation during both normal developmental and disease states. In the past decade, a number of methods have been developed and applied to characterize the genome-wide distribution of DNA methylation. Most of these methods endeavored to screen whole genome and turned to be enormously costly and time consuming for studies of the complex mammalian genome. Thus, they are not practical for researchers to study multiple clinical samples in biomarker research.

**Results:**

Here, we display a novel strategy that relies on the selective capture of target regions by liquid hybridization followed by bisulfite conversion and deep sequencing, which is referred to as liquid hybridization capture-based bisulfite sequencing (LHC-BS). To estimate this method, we utilized about 2 μg of native genomic DNA from YanHuang (YH) whole blood samples and a mature dendritic cell (mDC) line, respectively, to evaluate their methylation statuses of target regions of exome. The results indicated that the LHC-BS system was able to cover more than 97% of the exome regions and detect their methylation statuses with acceptable allele dropouts. Most of the regions that couldn't provide accurate methylation information were distributed in chromosomes 6 and Y because of multiple mapping to those regions. The accuracy of this strategy was evaluated by pair-wise comparisons using the results from whole genome bisulfite sequencing and validated by bisulfite specific PCR sequencing.

**Conclusions:**

In the present study, we employed a liquid hybridisation capture system to enrich for exon regions and then combined with bisulfite sequencing to examine the methylation statuses for the first time. This technique is highly sensitive and flexible and can be applied to identify differentially methylated regions (DMRs) at specific genomic locations of interest, such as regulatory elements or promoters.

## Background

The methylation of cytosines at CpG dinucleotides is an important regulatory modification in the somatic cells of mammals and other vertebrates [1,2]. It plays a vital role in regulating gene transcription during diverse biological processes, including embryonic development, X-chromosome inactivation, and the maintenance of pluripotency and chromosome stability [3-8]. Aberrant DNA methylation is associated with many common diseases. Recent evidence has demonstrated that the epigenetic silencing of tumour suppressor genes due to abnormal DNA hypermethylation is involved in cancer development and progression [8,9]. Due to its relative genomic stability and well-established role in cancer pathogenesis, it is likely that DNA methylation is an important biomarker for the diagnosis and prognosis of certain diseases [10].

Understanding the exact role of DNA methylation in normal developmental and disease states requires knowledge of the distribution of methylation in the genome. Fortunately, reference genome assemblies and massively parallel sequencing enable the high-resolution genome-wide profiling of cytosine methylation. With this profiling, it is possible to identify methylation biomarkers in clinical samples using adequately powered epigenome-wide association studies.

There are three main types of strategies that are currently available for the high-resolution detection of DNA methylation based on the next generation sequencing platform. The first is whole genome bisulfite sequencing (WGBS), in which sodium bisulfite is applied to the genomic samples to convert all unmethylated cytosines to uracils. The uracil is subsequently recognized as thymine after PCR amplification [11,12]. This technique is the gold standard for cytosine methylation analysis in both CpG and non-CpG contexts because it allows for methylation detection at a single-base resolution [11,12]. However, whole genome sequencing is extremely time consuming and costly, especially when used to screen complex genomes. For this reason, it is impractical for the survey of multiple biological samples.

The second approach is based on the enrichment of methylated DNA fragments such as MeDIP-seq [13,14] and MBD-seq [15], which employ 5-methylcytosine-specific antibodies and methyl-binding domain proteins, respectively, to precipitate methylated fractions from a randomly sheared genomic DNA sample. Although these two methods are able to enrich fragments containing 5-methylcytosine, they screen the whole genome without representation and cannot determine the methylation statuses of individual CpGs within the fragments [13-18]. Even when combined with bisulfite conversion, these methods were still hampered by the enrichment bias that was caused by the CpGs densities and methylation statuses of the DNA fragments [16].

The third approach includes reduced representation bisulfite sequencing (RRBS) and bisulfite padlock probes (BSPP) [19-21]. RRBS digests genomic DNA with a specific restriction enzyme followed by bisulfite conversion and sequencing of the size-selected fractions [19,20]. This technique is able to cover multiple regions, including CpG islands, promoters and enhancer elements, and can yield data at a single-base resolution. It is a robust and relatively low-cost method but is limited to profiling the defined regions that correspond to the recognition sites of certain restriction enzymes [17,18,20,22]. Theoretically, BSPPs could be applied to detect any genomic region of interest by the design of specific probes. However, because they are involved in the hybridization of bisulfite-converted genomic DNA, each probe can only target a limited number of CpGs. This limitation hampers the flexibility of the probe design and applicability of this technology [21].

In the present study, we designed a novel strategy, termed liquid hybridization capture-based bisulfite sequencing (LHC-BS), which utilized biotinylated RNA probes to capture target regions of native genomic DNA by liquid hybridization platform and then followed by sodium bisulfite treatment and next-generation sequencing to survey the methylation statuses of specific genomic regions. Exome capture sequencing is a widely used technique to study diseases, and methylation status is gaining recognition as an important topic [23]. Here, we performed target (exome) capturing and parallel bisulfite sequencing to test the reliability of our platform. To validate the applicability of our approach, two types of samples were used: the YanHuang peripheral blood sample (YH) and a mature dendritic cell (mDC) line. Our results demonstrated that the current LHC-BS method was as effective as the bisulfite system in detecting methylation statuses of the captured regions. The technique provided the foundations for future high-throughput examinations of the methylation statuses of any genomic regions of interest.

## Result

### Overview of LHC-BS

**Figure 1:**
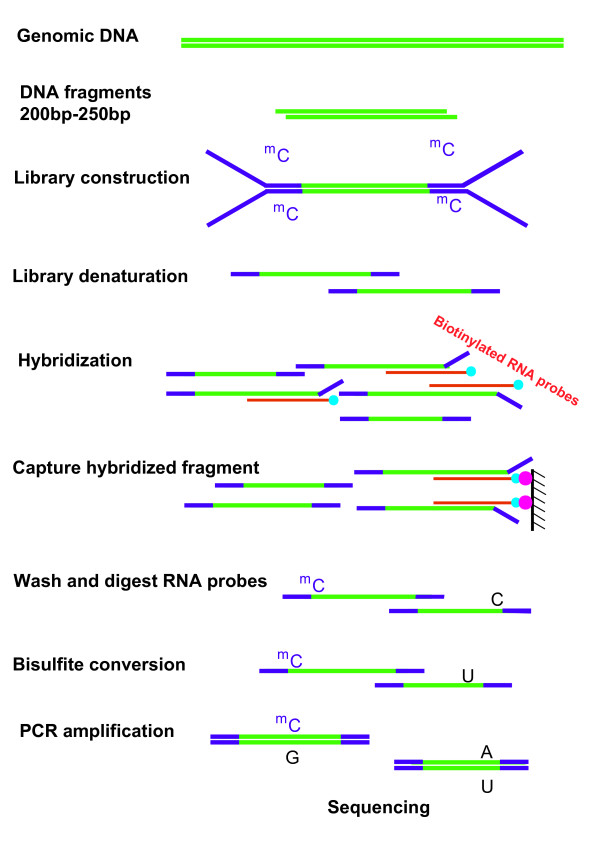
**Overview of the LHC-BS process**. Genomic DNA was fragmented, end-repaired and fitted with methylated adapters prior to the liquid hybridization. It was then subjected to bisulfite conversion and PCR amplification.

The LHC-BS assay was based on a protocol that applied predesigned biotinylated RNA probes to capture target genomic regions of interest for further analysis. In the current study, we used the Agilent SureSelect Human All Exon Kit to enrich all human exons, which totalled approximately 38 Mb. The capturing was achieved using a liquid hybridization system that required only 2-3 μg of genomic DNA for the library construction in contrast with the 20 μg that is required for the array-based capture method [24,25]. To briefly summarize the experimental procedures, 2-3 μg of genomic DNA were randomly fragmented to mean sizes of approximately 250 bp. The manufacturer's protocols were followed for creating the blunt ends and for the dA additions to the 3'ends and the methylcytosines modified adapter ligations. A total of 500 ng of each adapter-ligated library (147 ng/μl) were denatured and hybridised with biotinylated RNA probes at 65°C for 24 h. Then, Dynabeads^® ^M-280 Streptavidin was used for the separation of target fragments, after which the captured beads were washed and the DNA fragments were eluted. After the fragments were treated with sodium bisulfite, PCR amplification was performed and the amplicons were sequenced using the Illumina/HiSeq 2000 (Figure 1).

### Data generation

LHC-BS was successful in achieving sufficient coverage of the target regions based on a relatively small amount of sequencing. In this study, 88 M and 132 M raw reads were generated for the YH blood sample and mDC cell line, respectively (Additional File 1 Table S1). Of these reads, more than 75 M and 106 M reads were uniquely mapped back to the reference genome, giving unique map rates of 85.36% and 80.27%, respectively. On average, the depths of coverage were 58× and 63× for the YH and mDC exomes, respectively. We found that more than 97% of the target exons were covered by unique reads in both samples. Of these, 90.21% and 89.16% of the regions were covered by at least 10 reads for each sample, respectively. Furthermore, 71.93% of the reads were enriched in the target regions for the YH blood sample, and 75.96% for the mDC cell line, thus indicating the high capture specificity of this assay for exome.

Because bisulfite conversion can result in allele dropouts at low DNA concentrations, we further evaluated the potential rate of allele loss for the YH sample by evaluating heterozygous SNPs (more than 2 alleles) (unpublished). Totally, 7172 heterozygous alleles were identified in the target regions with sequencing depths of more than 10× in YH whole genome bisulfite sequencing research, and 6992 of them were identified heterozygous by the current designed method. These results indicated that the rate of allele dropout was 2.5% (Additional File 1 Table S2). The heterozygosity of A/G, C/T, G/A and C/T was not included in this analysis because it was possible that they were generated during the bisulfite conversion.

### Accuracy of target exon capture

**Figure 2:**
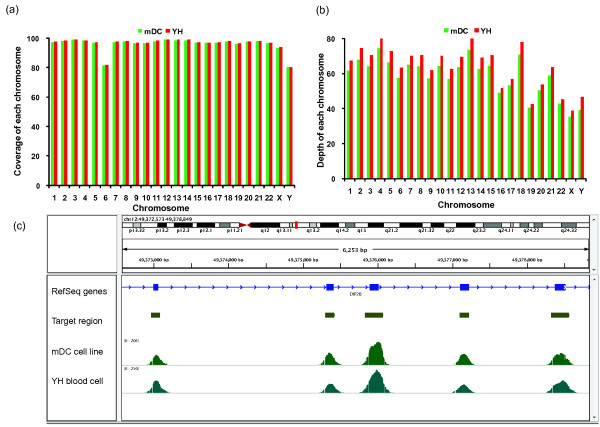
**Accuracy of LHC-BS for determining the methylation statuses of specific regions**. (a) The coverage of target regions (exons) from the YH and mDC genomes as captured by liquid hybridization using the LHC-BS platform that was based on uniquely mapped reads. (b) The sequencing depths of the exons that were captured from the YH and mDC genomic DNA. (c) Reads distributions that were derived from segments of the *DIP2B *gene originated from a portion of chromosome 12 (49,372,573- 49,378,849).

Based on the massive sequencing reads that were uniquely mapped to the reference genome, we further examined the read distribution across the whole genome. As indicated in Figures 2a and 2b, the reads were distributed across all chromosomes, indicating the efficient coverage of the target regions. The average sequencing depth for each chromosome was over 30× for the negative strands in both the YH and mDC samples. To estimate the accuracy of target capture, we randomly isolated data from a specific region of the *DIP2B *gene that is located on human chromosome 12 and used the Integrative Genomics Viewer (IGV) to assess the read distributions for both the YH and mDC samples. Using the IGV, we were able to determine that the profiles of the reads from the target region followed normal distributions, and most of them were enriched in the gene region (Figure 2c). These results verified the accuracy of this technique in the capture of target regions. The read distribution across chromosome 12 is presented in Additional File 2 Figure S1.

### Accuracy of methylation estimation of target regions

To estimate the methylation statuses of the captured exon regions and evaluate the efficacy of LHC-BS in detecting genomic DNA methylation, methylation levels were assessed based on the following equation for one certain cytosine site: mC reads/(C reads +mC reads)*100%. Considering that not all unmethylated cytosines could be converted to thymine following the bisulfite treatment and because it is believed that non-CpG methylation is barely discernable in human somatic cells [26], we estimated the conversion rate by calculating the methylation levels of the non-CpG sites and adjusted the methylation levels of the CpG sites accordingly. We had previously performed WGBS on both samples and obtained sufficient data to evaluate the genomic methylation statuses (unpublished data), and we were thus able to apply these data to estimate the accuracies of the methylation measurements that were obtained using the LHC-BS method. We isolated WGBS reads that represented exon regions with at least nine-fold depths of coverage and performed correlation analyses with the LHC-BS reads from both samples. A correlation coefficient of 0.907 was obtained for the YH blood sample from the comparison of data of the two methods (Additional File 3 Figure S2 a). Similarly, a correlation coefficient of 0.925 was reached for the comparison with the mDC cell line (Additional File 3 Figure S2 b). Such high consistencies between the two sets of data that were generated by the two distinct methods are indicative of the accuracy of LHC-BS because WGBS is considered to be the gold standard for measuring methylation.

**Figure 3:**
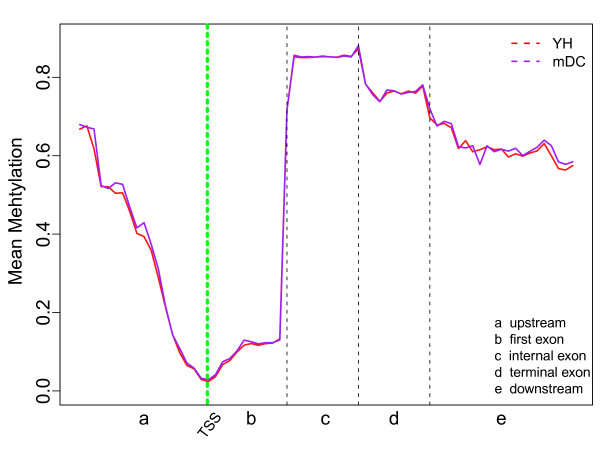
**Mean methylation levels of CG distributions in target regions**. The upstream region was defined as the 2 kb upstream of the TSS, and the downstream region was defined as the 2 kb downstream of the TTS. Data analysis results shown that sequencing depths were observed increase in areas that were closer to gene regions. Each region of the two parts was divided equally into 20 sections. Moreover, the first, internal and terminal exons each shared 10 sections. Each methylation level that was reported represented the mean value of the corresponding section.

Secondly, we addressed the methylation levels in all gene regions of the YH blood sample and mDC cell line. As indicated in Figure 3, the methylation levels across the target gene regions were very similar between the two samples, suggesting similarities between the two cell types that are both primarily comprised of immunocytes. Due to these parallels, we were able to examine technological reproducibility to some extent; specifically, the average methylation level of all CpGs in the genomic regions around the TSSs was identified to be 3%, which is consistent with the published results from the whole genome bisulfite sequencing of YH peripheral blood mononuclear cells [26].

To characterize the slight differences in the DNA methylation profiles between the samples, we performed pair-wise comparisons between the YH blood cells and mDC cell line. As expected, the results revealed that only 21 out of the 165637 targeted regions significantly differed between the two samples (see Methods), indicating highly similar DNA methylation profiles corresponding with the whole exon regions. A Gene Ontology (GO) analysis was employed to address the molecular functions of the genes from the 21exon regions that exhibited significantly different DNA methylation patterns (Table 1).

**Table 1:** Top 21 Gene Ontology categories between the YH and the mDC samples

Gene name	GO ID	NM	GO category	Methylation level of mDC	Methylation level of YH	*P *value
HELLS	GO:0005524	NM_018063	ATP binding	33.333	1.316	4.54E-03
MSI1	GO:0007399	NM_002442	nervous system development	20.833	2.632	8.23E-03
CEBPE	GO:0046983	NM_001805	protein dimerization activity	23.739	9.081	5.58E-79
EML2	GO:0007605	NM_012155	sensory perception of sound	27.621	13.554	9.63E-12
GLTSCR2	GO:0005634	NM_015710	nuclear	23.36	11.579	1.52E-04
TBC1D17	GO:0005096	NM_024682	GTPase activator activity	25.197	3.425	3.51E-14
BDH1	GO:0016491	NM_203314	oxidoreductase activity	22.892	11.345	6.49E-13
COL23A1	GO:0016021	NM_173465	integral to membrane	20.896	10.081	5.73E-03
WASF1	GO:0030041	NM_001024936	actin filament polymerization	22.215	10.467	9.32E-23
MYO1G	GO:0016459	NM_033054	myosin complex	21.907	8.239	5.16E-13
ARMCX2	GO:0016021	NM_014782	integral to membrane	31.395	14.286	5.12E-04
MMP21	GO:0008270	NM_147191	zinc ion binding	9.375	33.862	1.05E-07
ITGA7	GO:0016020	NM_002206	membrane	10.879	21.88	1.39E-11
DHRS12	GO:0016491	NM_024705	oxidoreductase activity	8.696	23.144	1.55E-05
AURKC	GO:0016740	NM_001015878	transferase activity	10.256	30.769	9.17E-03
SOX18	GO:0006357	NM_018419	regulation of transcription from RNA polymerase II promoter	3.656	20.225	2.55E-15
CIDEC	GO:0006917	NM_022094	induction of apoptosis	8.333	20.098	6.22E-04
PF4	GO:0045653	NM_002619	negative regulation of megakaryocyte differentiation	0	22.222	3.13E-03
MARCKS	GO:0051015	NM_002356	actin filament binding	5.405	30.088	4.66E-03
PRAF2	GO:0016021	NM_007213	integral to membrane	8.333	26.02	3.00E-03
FAM155B	GO:0016021	NM_015686	integral to membrane	0	21.053	4.02E-04

The methylation level represents the average methylation level of the CpGs in the region. The *P *value was calculated using the chi-square test.

### Validation of DNA methylation by BS-PCR

**Figure 4:**
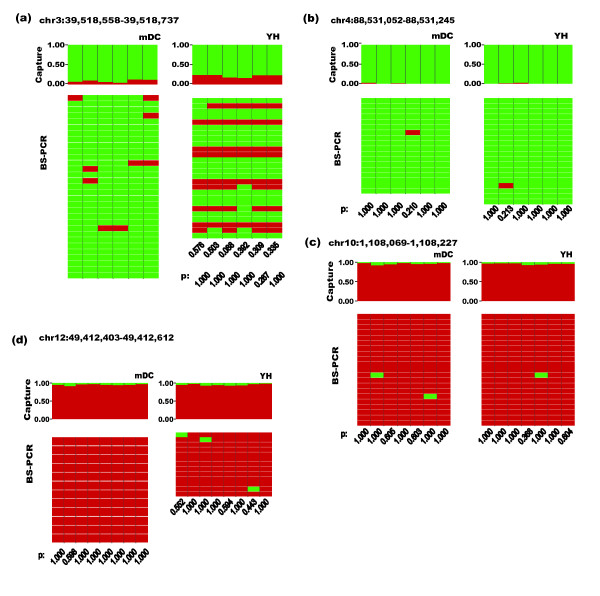
**Comparison of methylation levels between LHC-BS and BS-PCR**. The top of the figure represents the methylation levels of CpGs in the YH blood and mDC samples that were detected by LHC-BS. The height of the red box represents the methylation level of a CpG site. Each rectangle underneath represents the methylation status of a CG in a fragment that was detected by BS-PCR. Red represents a methylated CpG site, and green represents a nonmethylated CpG site. The *P *values were calculated using the chi-square or Fisher's tests. (a) Comparison of the methylation levels that were detected in a moderately methylated region of the 5'UTR of the *MOBP *gene, (b) a mildly methylated region of the *DSPP *gene and two highly methylated regions of the *WDR37 *(c) and *DIP2B *(d) genes using either HLC-BS or BS-PCR.

Certain biases may be introduced during exome capture, thus hampering the accuracy of LHC-BS. To confirm the methylation events that were detected by LHC-BS, BS-PCR was performed on four randomly chosen genomic fragments. These fragments had low, moderate or high methylation levels in both the YH blood sample and mDC cell line, and there were 27 individual CpG sites in total. Fisher's tests were applied to verify statistical efficacy, and it was observed that none of the CpG sites presented with significant differences in DNA methylation levels when the LHC-BS and BS-PCR results were compared (Additional File 1 Table S3). For instance, a region that is located in the 5'UTR of the *MOBP *gene was identified by LHC-BS to have moderate methylation levels of 9.80% and 22.37% in the mDC cell line and YH blood sample, respectively. Similar results were observed for the two samples following BS-PCR (Figure 4a). In the other three verified regions, sequences from the *DSPP *gene presented with nearly undetectable methylation levels (Figure 4b), and the *WDR37 *and *DIP2B *genes were shown to be heavily methylated using both technologies (Figure 4c and 4d). Thus, taking into account the random selection of the DNA fragments for validation, we did not observe any bias in the detection of methylation levels using LHC-BS.

## Discussion

Along with the completion of various genome sequencing projects and more wide spread uses of high-throughput sequencing technologies, many strategies have been developed to assess DNA methylation that require the enrichment of specific genomic regions of interest. For example, microarray-based strategies utilized bisulfite-converted DNA to probe defined regions by the bimodal hybridization of either unconverted or converted probes [24]. This technique enables the detection of original methylation states at specific CpG sites, but it is difficult to design an adequate number of unique probes to extend this approach to a genome-wide scale. However, Hodges *et al. *[25] recently modified this strategy and integrated it with deep sequencing. For each CpG island, they designed two sets of probes; one designed to detect no conversion at any of the CpG residues and another to distinguish the full conversion of CpGs to TpGs. They hypothesized that even with completely random CpG methylation patterns, only half of the CpG sites within a given probe would contribute a mismatch, and that was tolerated. However, the methods that were based on microarray hybridization enrichment were either laborious or required large volumes of DNA and were therefore not easily adapted to the massive screening of clinical samples. In the current study, we described a novel technique that combined sequence capture with bisulfite conversion and deep sequencing. In contrast to the strategy that Hodges *et al. *described [25], we utilized biotinylated RNA probes without bisulfite conversion to capture native genomic regions of interest. This adjustment in the experimental procedures allowed for a drastic decrease in the amount of DNA that was required for capture and more accurate measurements of methylation statuses. Previously, Hodges *et al. *used 20 μg of PCR-amplified genomic DNA for a microarray-based capture experiment. In contrast, 2-3 μg of genomic DNA was used in the library construction for the present study, and 500 ng of the constructed library was sufficient for the hybridization capture and subsequent bisulfite treatment. To protect the captured library, 200 ng of sheared λDNA was added to act as carriers of the DNA that had been eluted from the liquid capture in the current bisulfite treatment process. Based on these modifications, we were not only able to facilitate the probe design process by not requiring any prior consideration of methylation sites but were also able to utilize small amounts of native, unamplified input DNA to examine the methylation statuses of specific regions by designing probes that covered different genomic regions.

Recent investigations involving DNA methylation within different gene regions showed that exons are disproportionately enriched in densely methylated genomic elements [21,26-29]. Hypermethylation that occurs downstream of the TSS and first exon is closely associated with transcriptional silencing, whereas methylation that occurs in the more downstream portions of the gene body is not [26,29]. In highly expressed genes, the promoter regions are hypomethylated while the rest of the gene-body regions are considerably methylated. However, weakly expressed genes are moderately methylated in both the promoter and gene-body regions. Thus, the function of intragenic DNA methylation is ambiguous. Previous research has indicated that intragenic DNA methylation, exonic nucleosomes and histone modifications may function together to regulate transcript splicing and gene expression [23,30,31].

Here, we profiled the human whole exome methylation statuses of two samples and obtained the same patterns as were previously detected using WGBS [26]. Using this set of probes, we covered 31% of the promoter regions and approximately 100% of the exons. Most of the regions that were not covered (Figure 2a) were distributed in chromosomes 6 and Y. Because we only calculated uniquely mapped reads, we determined that a large number of multiple mapping were present in the uncovered regions of chromosomes 6 and Y. For this reason, the data that was obtained using LHC-BS did not provide accurate methylation information for these regions (Additional File 1 Table S4). Additionally, to collect sufficient information, we generated massive amounts of data using excessive sequencing depths for the two samples, which reasonably led to the high duplication rate of 40.32% for the YH blood sample and 61.04% for the mDC cells. However, this value can be adjusted by reducing the amount of raw sequencing data in future studies. It has been reported that bisulfite conversion may result in allele dropouts at low DNA concentrations [32]. We calculated the allele loss rate to be 2.5%, indicating efficient allele enrichment.

We applied a commercially available liquid hybridization system for exon capture, for which all of the probes were designed using the Watson strand of the human genome. Therefore, only information regarding the methylation of the Crick strand was obtained in this study. However, DNA methylation occurs primarily at CpG dinucleotides in mammalian genomes [1,2,33]. For each round of DNA replication, DNMT1 DNA methyltransferase, which has a preference for hemimethylated DNA, fills in the missing methylation sites on the newly synthesized strand [34-36]. Thus, the presence of symmetric CpG methylation was presumed for the two DNA strands composing each human chromosome. For non-CpG methylation, which plays an important role in maintaining the pluripotency of stem cells [11], we may design probes in future studies that cover the double-stranded regions of interest.

## Conclusions

In the present study, we applied a liquid hybridisation capture system to enrich for specific genomic regions, which was combined with bisulfite sequencing to examine the methylation statuses of the human exome for the first time. Two different samples were profiled, and the methylation patterns of the exons were compared with methylation patterns that had been obtained using WGBS. These comparisons demonstrated the high accuracy of methylation estimation that is possible using this novel LHC-BS method. In contrast with the array-based capture strategy [25], we captured target regions without pre-converting genomic DNA using a bisulfite treatment. Instead, we used 2-3 μg of genomic DNA to construct a library and 500 ng of them was then subjected to target regions capture and a following bisulfite treatment. This modification improved the efficacy of this platform for methylation profiling, especially with regard to its cost-effective use in the analysis of multiple clinical samples for biomarker detection.

## Methods

### Sample preparation and DNA library construction

Two types of genomic DNA were isolated from a peripheral blood sample that was obtained from the same individual whose genome and methylome had been previously sequenced (YH) [26,37] and from a mature human dendritic cell line (mDC). The total DNA from the peripheral blood sample was prepared using the QIAamp DNA Blood Mini Kit following the manufacturer's instructions, and the DNA from the mature human dendritic cells was isolated by proteinase K digestion and phenol/chloroform extraction.

Prior to the library construction, 2-3 μg of genomic DNA from each sample was fragmented using a Covarias sonication system to mean sizes of approximately 200 bp. After fragmentation, libraries were constructed according to the Illumina Paired-End protocol. Briefly, the purified, randomly fragmented DNA was treated with a mix of T4 DNA polymerase, Klenow fragments, T4 polynucleotide kinase and a nucleotide triphosphate mix to repair the ends by blunting and phosphorylation. The blunted DNA fragments were subsequently 3'-adenylated using the Klenow fragment (3'-5' exo-) and ligated by T4 DNA ligase to BGI-designed PE Index Adaptors that had been synthesized with 5'-methylcytosine in place of cytosine. After each step, the DNA was purified using the QIAquick PCR Purification Kit (Qiagen). The constructed libraries were stored at -20°C until the next step of hybridization.

### Liquid hybridization-based exon capture

The SureSelect Human All Exon 38 Mb Kit was used for the hybridization process. The probes nearly covered all of the exons that were annotated by the CCDS in September of 2008. The constructed libraries for the capture were quantified using the Quant-iT dsDNA HS Assay Kit with the Invitrogen Qubit fluorometer. Five hundred nanograms of each library at concentrations of 147 ng/μl were required for the hybridizations as described in the protocol for the SureSelect Target Enrichment System for an Illumina Paired-End Sequencing Library. The hybridizations were performed using a thermal cycler that was set at 65°C for 24 hours with the lid heated to 105°C; then, Dynabeads^® ^M-280 Streptavidin (Invitrogen) was used to capture the biotinylated RNA probes that had hybridized with the fragments of the target regions. Finally, the captured library samples were washed and purified for the bisulfite conversion.

### Bisulfite conversion and PCR amplification

The bisulfite conversion of the captured exon DNA was performed according to the instructions for the ZYMO EZ DNA Methylation-Gold Kit™ with some modifications. A total of 200 ng of fragmented λDNA were added to act as carriers for the elution products from the hybridization. After the samples were denatured and treated with sodium bisulfite, they were desulfonated, and the cytosine was converted to uracil. PCR was carried out in a final reaction volume of 50 μl that included 10 μl eluted bisulfite conversion products, 4 μl 2.5 mM dNTP, 5 μl 10× buffer, 0.5 μl JumpStart™ Taq DNA Polymerase, 2 μl PCR primers and 28.5 μl water. The thermal cycling program was as follows: 94°C for 1 min, 18 cycles of 94°C for 10 s, 58°C for 30 s, 72°C for 30 s and a final 5 min incubation at 72°C. The temperature was held at 12°C following the termination of the cycling program. PCR products that ranged in size from 200 to 400 bp were selected and gel purified using the QIAquick Gel Extraction Kit (Qiagen). They were then analyzed by the Bioanalyser Analysis System (Agilent, Santa Clara, USA) and quantified using real time PCR. According to the real time PCR measurements, two PCR products of distinct samples were pooled on one lane and sequenced by the Illumina Hiseq2000 using 90 bp paired-end sequencing reads.

### Bisulfite sequencing of specific regions

Specific regions of bisulfite-treated genomic DNA from each of the samples were PCR amplified, and the products were cloned and sequenced using conventional Sanger sequencing. Briefly, 500 ng of genomic DNA from each sample were converted according to the ZYMO EZ DNA Methylation-Gold Kit™ manufacturer's instructions. PCR primers were designed using the online MethPrimer software http://www.urogene.org/methprimer/index.html. The PCR primer information is listed in Additional File 1 Table S5. These primers were designed to recognize regions that lack CpG sites to avoid any amplification bias that may have occurred due to the differences between the methylated and unmethylated sequences. Thermal cycling was performed as follows: 94°C for 1 min, 30 cycles of 94°C for 10 s, 58°C for 30 s, 72°C for 30 s and a final 5 min incubation at 72°C. The temperature was held at 12°C following the termination of the cycling program. The PCR products were purified using the QIAquick Gel Extraction Kit (Qiagen). The purified PCR products were subcloned, and colonies from each region were sequenced using the 3730 Genetic Analyzer (Applied Biosystems) to assess the methylated cytosine levels.

### Bioinformatic analysis

The captured fragments were sequenced using an Illumina Hiseq2000 sequencer that generated raw data in a 90 bp paired-end Fastq format. The data were processed using the Illumina base-calling pipeline. Subsequently, all reads were aligned to the hg18 reference genome using the SOAP2.01 aligner [38]. Briefly, we used the human reference genome to derive the soap libraries for the assessment of the mapping results of the bisulfite treatment. In the mapping process, the seed of a read was 30 bp, and 2 mismatches were permitted. Only mismatches of 5 bp were allowed in the total read. Methylcytosines were identified according to a previously published strategy [26]. To reduce any bias that resulted from the PCR amplification, we chose optimal reads that possessed fewer gaps and mismatches when more than one read was mapped to the same position. Additionally, we preferred the paired-end mapped reads. Methylation levels were calculated using the following formula for certain cytosine types: mC reads/(C reads +mC reads)*100%. Because not all of the unmethylated cytosines could be converted to uracils by the bisulfite treatment, we estimated the non-conversion rate using the methylation rate of non-CpG sites.

To validate the results of the LHC-BS, Pearson's correlation analysis were utilized to conduct pair-wise comparisons between datasets that were derived from the LHC-BS and WGBS assays. For the BS-PCR validation analysis, the BLAST algorithm was applied to align the sequences (expect value = 1E-10). The Fisher's test was used to statistically define the significances of the differences between the methylation information that was generated by LHC-BS and BS-PCR (*P *value < 0.01).

Three parameters were applied to identify the differentially methylated regions between the mDC cell line and YH blood sample. First, we summed all of the methylated and unmethylated nucleotides in the CpG sites for each region of the two samples, respectively. The Fisher's test was used to analyze the methylation differences for each target region using a *P *value < 0.01. Second, we separated out the regions that showed over two-fold increases in methylation levels between samples. Third, considering the non-uniformity of the methylation levels, we selected regions with differences in methylation of over 20% between the two samples. The obtained differentially methylated regions were then examined using the BGI WEGO software to determine the functional information for their respective genes.

## Authors' contributions

XZ, HJ, JS, and JW conceived and designed the present study; HL, JW, RW, and JW performed the experiments; GJ analyzed the data; JW, FG, GJ, MW, and HJ wrote the manuscript. All authors read and approved the final manuscript.

## Supplementary Material

Additional file 1 **Supplementary Tables 1-5. Table S1: **Data statistics of YH and mDC from HLC-BS. **Table S2: **Assessment of the dropout of alleles of heterozygous genes from the YH LHC-BS data. **Table S3: **Statistics of the methylation statuses of 27 individual CpG sites that were analyzed by HLC-BS and BS-PCR. *P *values were calculated using the chi-square or Fisher's tests. **Table S4: **Analysis of the undetected target region characteristics. **Table S5: **BS-PCR primer information. Click here for file

Additional file 2 **Figure S1 LHC-BS read distribution along chromosome 12**. Click here for file

Additional file 3 **Figure S2 Comparison of methylation rates between WGBS and LHC-BS**. (a) The Pearson's correlation coefficient from the YH blood sample was 0.907, and the confidence interval was 0.902-0.912; (b) for the mDC cell line, the correlation coefficient was 0.925, and the confidence interval was 0.921-0.928. The y-axis shows the methylation rate of a cytosine as determined by the whole genome sequencing of bisulfite-treated DNA, and the x-axis shows the methylation level as determined by HLC-BS. This analysis was restricted to cytosines with at least nine reads in both samples. Click here for file

## References

[B1] Gama-Sosa MA Slagel VA Githens S Kuo KC Ehrlich M Tissue-specific differences in DNA methylation in various mammals Biochim Biophys Acta 1983 740 212 219 10.1016/0167-4781(83)90079-9 6860672

[B2] Bird A DNA methylation patterns and epigenetic memory Genes Dev 2002 16 6 21 10.1101/gad.947102 11782440

[B3] Li E Bestor TH Jaenisch R Targeted mutation of the DNA methyltransferase gene results in embryonic lethality Cell 1992 69 915 926 10.1016/0092-8674(92)90611-f 1606615

[B4] Okano M Bell DW Haber DA Li E DNA methyltransferases Dnmt3a and Dnmt3b are essential for de novo methylation and mammalian development Cell 1999 99 247 257 10.1016/s0092-8674(00)81656-6 10555141

[B5] Reik W Stability and flexibility of epigenetic gene regulation in mammalian development Nature 2007 447 425 432 10.1038/nature05918 17522676

[B6] Heard E Disteche CM Dosage compensation in mammals: fine-tuning the expression of the X chromosome Genes Dev 2006 20 1848 1867 10.1101/gad.1422906 16847345

[B7] Lippman Z Gendrel AV Black M Vaughn MW Dedhia N Lavine K Mittal V May B Carrington JC Doerge RW Colot V Martienssen R Role of transposable elements in heterochromatin and epigenetic control Nature 2004 430 471 476 10.1038/nature02651 15269773

[B8] Robertson KD DNA methylation and human disease Nat Rev Genet 2005 6 597 610 10.1038/nrg1655 16136652

[B9] Feinberg AP Phenotypic plasticity and the epigenetics of human disease Nature 2007 447 433 440 10.1038/nature05919 17522677

[B10] Bock C Epigenetic biomarker development Epigenetics 2009 1 99 110 10.2217/epi.09.6 22122639

[B11] Lister R Pelizzola M Dowen RH Hawkins RD Hon G Tonti-Filippini J Nery JR Lee L Ye Z Ngo QM Edsall L Antosiewicz-Bourget J Stewart R Ruotti V Millar AH Thomson JA Ren B Ecker JR Human DNA methylomes at base resolution show widespread epigenomic differences Nature 2009 462 315 322 10.1038/nature08514 PMC2857523 19829295

[B12] Feng S Zhang X Chen Z Merriman B Haudenschild CD Pradhan S Nelson SF Pellegrini M Jacobsen SE Shotgun bisulphite sequencing of the Arabidopsis genome reveals DNA methylation patterning Nature 2008 452 215 219 10.1038/nature06745 PMC2377394 18278030

[B13] Keshet I Schlesinger Y Farkash S Rand E Hecht M Segal E Pikarski E Young RA Niveleau A Cedar H Simon I Evidence for an instructive mechanism of de novo methylation in cancer cells Nat Genet 2006 38 149 153 10.1038/ng1719 16444255

[B14] Jacinto FV Ballestar E Esteller M Methyl-DNA immunoprecipitation (MeDIP): hunting down the DNA methylome Biotechniques 2008 44 35 43 10.2144/000112708 18254377

[B15] Serre D Lee BH Ting AH MBD-isolated Genome Sequencing provides a high-throughput and comprehensive survey of DNA methylation in the human genome Nucleic Acids Res 2010 38 391 399 10.1093/nar/gkp992 PMC2811030 19906696

[B16] Li N Ye M Li Y Yan Z Butcher LM Sun J Han X Chen Q Zhang X Wang J Whole genome DNA methylation analysis based on high throughput sequencing technology Methods 2010 52 203 212 10.1016/j.ymeth.2010.04.009 20430099

[B17] Bock C Brinkman AB Müller F Simmer F Gu H Jäger N Gnirke A Stunnenberg HG Meissner A Quantitative comparison of genome-wide DNA methylation mapping technologies Nat Biotechnol 2010 28 1106 1114 10.1038/nbt.1681 PMC3066564 20852634

[B18] Harris RA Wang T Coarfa C Nagarajan RP Hong C Downey SL Johnson BE Fouse SD Delaney A Zhao Y Olshen A Ballinger T Zhou X Forsberg KJ Gu J Echipare L O'Geen H Lister R Pelizzola M Xi Y Epstein CB Bernstein BE Hawkins RD Ren B Chung WY Gu H Bock C Gnirke A Zhang MQ Haussler D Comparison of sequencing-based methods to profile DNA methylation and identification of monoallelic epigenetic modifications Nat Biotechnol 2010 28 1097 1105 10.1038/nbt.1682 PMC2955169 20852635

[B19] Meissner A Gnirke A Bell GW Ramsahoye B Jaenisch R Reduced representation bisulfite sequencing for comparative high-resolution DNA methylation analysis Nucleic Acids Res 2005 33 5868 5877 10.1093/nar/gki901 PMC1258174 16224102

[B20] Smith ZD Gu H Bock C Gnirke A Meissner A High-throughput bisulfite sequencing in mammalian genomes Methods 2009 48 226 232 10.1016/j.ymeth.2009.05.003 PMC2864123 19442738

[B21] Ball MP Li JB Gao Y Lee JH Park IH Xie B Daley GQ Church GM Targeted and genome-scale strategies reveal gene-body methylation signatures in human cells Nat Biotechnol 2009 27 361 368 10.1038/nbt.1533 PMC3566772 19329998

[B22] Gu H Bock C Jäger N Smith ZD Tomazou E Gnirke A Lander ES Meissner A Genome-scale DNA methylation mapping of clinical samples at single-nucleotide resolution Nat Methods 2010 7 133 136 10.1038/nmeth.1414 PMC2860480 20062050

[B23] Maunakea AK Nagarajan RP Bilenky M Ballinger TJ D'Souza C Fouse SD Johnson BE Hong C Nielsen C Zhao Y Turecki G Delaney A Varhol R Thiessen N Shchors K Heine VM Rowitch DH Xing X Fiore C Schillebeeckx M Jones SJ Haussler D Marra MA Hirst M Wang T Costello JF Conserved role of intragenic DNA methylation in regulating alternative promoters Nature 2010 466 253 257 10.1038/nature09165 PMC3998662 20613842

[B24] Beck S Rakyan VK The methylome: approaches for global DNA methylation profiling Trends Genet 2008 24 231 237 10.1016/j.tig.2008.01.006 18325624

[B25] Hodges E Smith AD Kendall J Xuan Z Ravi K Rooks M Zhang MQ Ye K Bhattacharjee A Brizuela L Wigler M Hannon GJ Hicks JB High definition profiling of mammalian DNA methylation by array capture and single molecule bisulfite sequencing Genome Res 2009 19 1593 1605 10.1101/gr.095190.109 PMC2752124 19581485

[B26] Li Y Zhu J Tian G Li N Li Q Ye M Zheng H Yu J Wu H Sun J Zhang H Chen Q Luo R Chen M He Y Jin X Zhang Q Yu C Zhou G Sun J Huang Y Zheng H Cao H Zhou X Guo S Hu X Li X Kristiansen K Bolund L Xu J The DNA methylome of human peripheral blood mononuclear cells PLoS Biol 2010 8 e1000533 10.1371/journal.pbio.1000533 PMC2976721 21085693

[B27] Hellman A Chess A Gene body-specific methylation on the active X chromosome Science 2007 315 1141 1143 10.1126/science.1136352 17322062

[B28] Suzuki MM Bird A DNA methylation landscapes: provocative insights from epigenomics Nat Rev Genet 2008 9 465 476 10.1038/nrg2341 18463664

[B29] Brenet F Moh M Funk P Feierstein E Viale AJ Socci ND Scandura JM DNA methylation of the first exon is tightly linked to transcriptional silencing PLoS One 2011 6 e14524 10.1371/journal.pone.0014524 PMC3022582 21267076

[B30] Chodavarapu RK Feng S Bernatavichute YV Chen PY Stroud H Yu Y Hetzel JA Kuo F Kim J Cokus SJ Casero D Bernal M Huijser P Clark AT Krämer U Merchant SS Zhang X Jacobsen SE Pellegrini M Relationship between nucleosome positioning and DNA methylation Nature 2010 466 388 392 10.1038/nature09147 PMC2964354 20512117

[B31] Choi JK Contrasting chromatin organization of CpG islands and exons in the human genome Genome Biol 2010 11 R70 10.1186/gb-2010-11-7-r70 PMC2926781 20602769

[B32] Ito Y Koessler T Ibrahim AE Rai S Vowler SL Abu-Amero S Silva AL Maia AT Huddleston JE Uribe-Lewis S Woodfine K Jagodic M Nativio R Dunning A Moore G Klenova E Bingham S Pharoah PD Brenton JD Beck S Sandhu MS Murrell A Somatically acquired hypomethylation of IGF2 in breast and colorectal cancer Hum Mol Genet 2008 17 2633 2643 10.1093/hmg/ddn163 PMC2515372 18541649

[B33] Jones PA The DNA methylation paradox Trends Genet 1999 15 34 37 10.1016/s0168-9525(98)01636-9 10087932

[B34] Bestor TH Ingram VM Two DNA methyltransferases from murine erythroleukemia cells: purification, sequence specificity, and mode of interaction with DNA Proc Natl Acad Sci USA 1983 80 5559 5563 10.1073/pnas.80.18.5559 PMC384297 6577443

[B35] Hermann A Goyal R Jeltsch A The Dnmt1 DNA-(cytosine-C5)-methyltransferase methylates DNA processively with high preference for hemimethylated target sites J Biol Chem 2004 279 48350 48359 10.1074/jbc.M403427200 15339928

[B36] Wu SC Zhang Y Active DNA demethylation: many roads lead to Rome Nat Rev Mol Cell Biol 2010 11 607 620 10.1038/nrm2950 PMC3711520 20683471

[B37] Li G Ma L Song C Yang Z Wang X Huang H Li Y Li R Zhang X Yang H Wang J Wang J The YH database: the first Asian diploid genome database Nucleic Acids Res 2009 37 D1025 1028 10.1093/nar/gkn966 PMC2686535 19073702

[B38] Li R Li Y Kristiansen K Wang J SOAP: short oligonucleotide alignment program Bioinformatics 2008 24 713 714 10.1093/bioinformatics/btn025 18227114

