# Supplementary figures and images for: High resolution profiling of human exon methylation by liquid hybridization capture-based bisulfite sequencing
Source: BMC Genomics. 2011 Dec 8;12:597. doi: 10.1186/1471-2164-12-597 (PMC3295804; doi:10.1186/1471-2164-12-597)

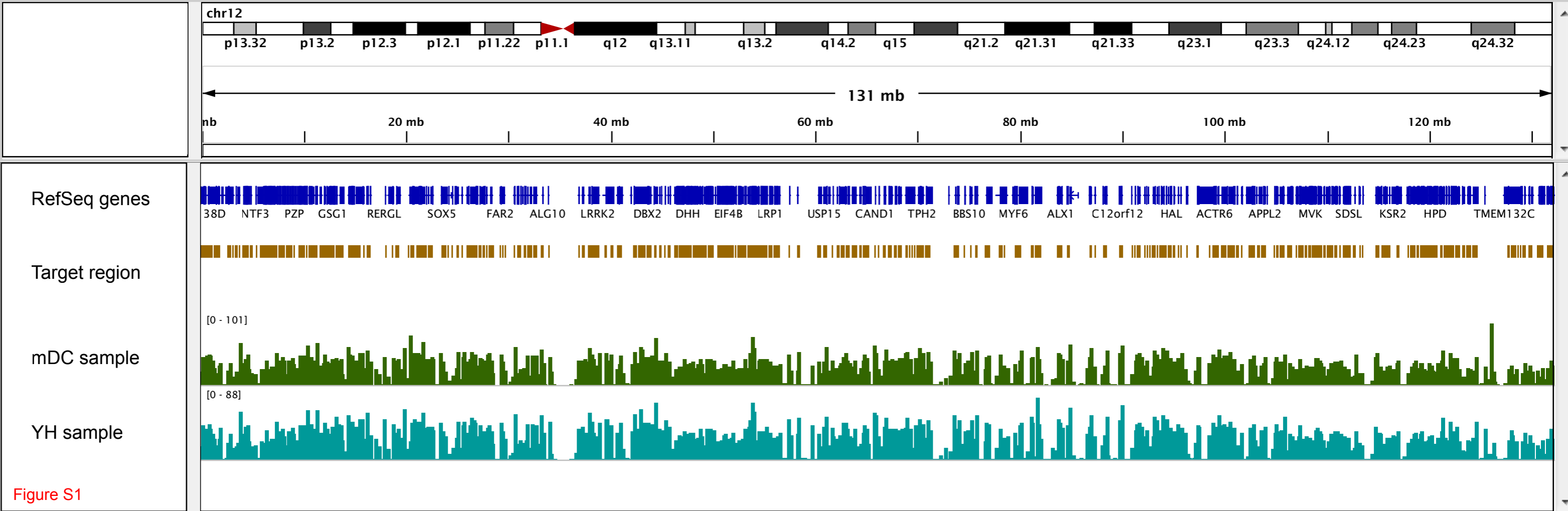

Supplement: Additional file 2 — Figure S1 LHC-BS read distribution along chromosome 12. [file 1471-2164-12-597-S2.PDF]

(a) Pearson Correlation=0.907 (YH)

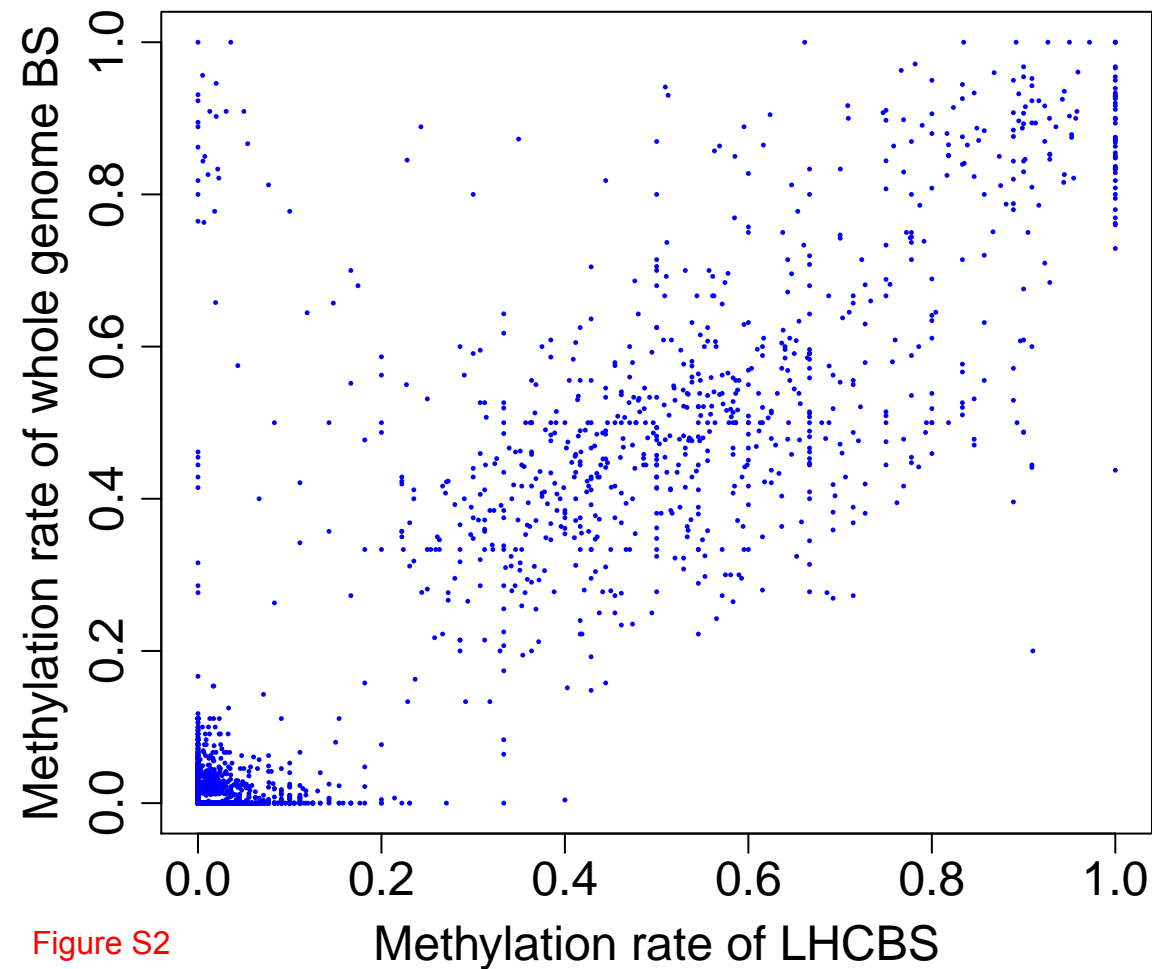

(b) Pearson Correlation=0.925 (mDC)

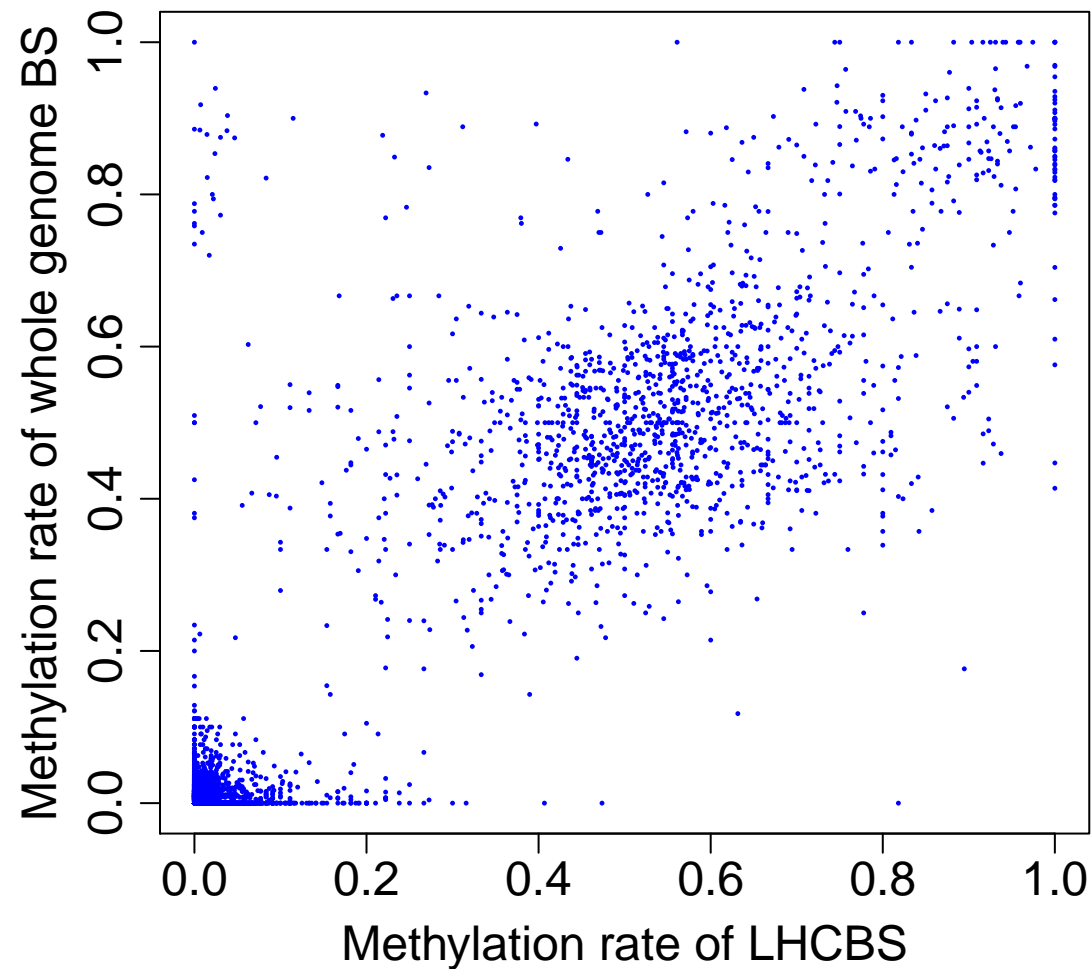

Supplement: Additional file 3 — Figure S2 Comparison of methylation rates between WGBS and LHC-BS. (a) The Pearson's correlation coefficient from the YH blood sample was 0.907, and the confidence interval was 0.902-0.912; (b) for the mDC cell line, the correlation coefficient was 0.925, and the confidence interval was 0.921-0.928. The y-axis shows the methylation rate of a cytosine as determined by the whole genome sequencing of bisulfite-treated DNA, and the x-axis shows the methylation level as determined by HLC-BS. This analysis was restricted to cytosines with at least nine reads in both samples. [file 1471-2164-12-597-S3.PDF]
